# The Effects of Aerobic Exercise on the Recovery of Walking Ability and Neuroplasticity in People with Multiple Sclerosis: A Systematic Review of Animal and Clinical Studies

**DOI:** 10.1155/2017/4815958

**Published:** 2017-10-17

**Authors:** Augustine Joshua Devasahayam, Matthew Bruce Downer, Michelle Ploughman

**Affiliations:** The Recovery and Performance Laboratory, Faculty of Medicine, Memorial University of Newfoundland, Room 400, L.A. Miller Centre, 100 Forest Road, St. John's, NL, Canada A1A 1E5

## Abstract

**Introduction:**

Walking is of high priority for people with multiple sclerosis (PwMS). It remains unclear whether aerobic exercise can improve walking ability and upregulate neurotrophins. This review aims to consolidate evidence to develop optimal aerobic training parameters to enhance walking outcomes and neuroplasticity in PwMS.

**Methods:**

Clinical studies examining aerobic exercise for ≥3 weeks, having outcomes on walking with or without neurotrophic markers, were included. Studies utilizing animal models of MS were included if they employed aerobic exercise with outcomes on neurological recovery and neurotrophins. From a total of 1783 articles, 12 clinical and 5 animal studies were included.

**Results:**

Eleven clinical studies reported improvements in walking ability. Only two clinical studies evaluated both walking and neurotrophins, and neither found an increase in neurotrophins despite improvements in walking. Patients with significant walking impairments were underrepresented. Long-term follow-up revealed mixed results. Two animal studies reported a positive change in both neurological recovery and neurotrophins.

**Conclusion:**

Aerobic exercise improves walking ability in PwMS. Gains are not consistently maintained at 2- to 9-month follow-up. Studies examining levels of neurotrophins are inconclusive, necessitating further research. Aerobic exercise enhances both neurological recovery and neurotrophins in animal studies when started 2 weeks before induction of MS.

## 1. Introduction

Multiple sclerosis (MS) is a demyelinating autoimmune disease affecting approximately 2.3 million people worldwide [1]. Improved healthcare has led to people living longer with MS and disease-modifying drugs have helped more patients remain stable in their disease [2–5]. However, relapses and slow decline of function still occur over time and most people with MS (PwMS) will develop permanent physical disability [1–5]. The rehabilitative approach to MS has primarily focused on teaching compensation for physical impairments rather than fostering neuroplasticity and recovery of function [6, 7]. Recent research suggests that neuroplasticity does occur among PwMS [8] and there may be more opportunities for recovery after relapse than was previously believed [9].

Walking is of high priority for PwMS [10] and there is a need to develop effective treatments to mitigate the progressive difficulty in walking experienced by PwMS [11, 12]. Ideally, rehabilitative interventions should maximize walking ability, while simultaneously facilitating plasticity of neural pathways that execute walking to foster long-term restoration of function [13–15]. Although the exact cellular cascades underlying the neural plasticity for walking remain to be explored, there is a general consensus suggesting that such plasticity may take place involving neuroplastic markers at the site of injury and/or lesions [16, 17].

Aerobic exercise is one intervention that has potential to affect multiple underlying targets such as enhancing markers of neuroplasticity, attenuating neural inflammation, and improving tolerance for physical activity, and because of reciprocal limb movements, it also helps restore walking ability [14, 15]. Evidence suggests that aerobic exercise promotes neuroplasticity by upregulating neurotrophins such as brain derived neurotrophic factor (BDNF), nerve growth factor (NGF), neurotrophin-3 (NT3), and neurotrophin-4 (NT4) [18–20]. Among these, BDNF has been thought to have great potential as a therapeutic agent due to its ability to cross the blood-brain barrier (BBB) [21]. There is, however, a report that, even in the presence of a pronounced BBB disruption, there are no significant increases in plasma BDNF levels [22]. Nevertheless, BDNF is suggested to play a central role in neuroplasticity as well as exercise-induced enhancement in learning and memory [23, 24].

The regulation of neurotrophic factors has been implicated in the repair of neural structures damaged by the demyelination process, resulting in functional recovery in PwMS [25]. Current literature suggests that a single exercise bout and/or long-term training could transiently increase BDNF synthesis and induce a cascade of neurotrophic and neuroprotective effects [19]. Recent research has reported that an acute bout of exercise could alter BBB permeability [26], which in turn could result in larger BDNF release after a few weeks of training (possibly through repeated spells of altered BBB permeability). In line with this view, the meta-analysis by Dinoff et al. [27] concluded that regular aerobic training for ≥2 weeks elevated resting BDNF levels. Therefore, a familiar functional task such as walking could be incorporated as an aerobic exercise, elevating BDNF levels and fostering long-term improvements on walking performance among PwMS. Wens et al. [28] explored this idea by studying the effects of a 24-week combined training program that included cardiovascular treadmill training and reported significant increases in circulating BDNF and exercise tolerance on a seated bike test among persons with relapsing-remitting MS. However, it is unclear whether such aerobic-type training could increase both BDNF levels and neuroplasticity required for walking in PwMS [28], forming the basis of this review. Furthermore, the exact exercise parameters to evoke change in walking ability (while upregulating neurotrophins) in terms of FITT (frequency, intensity, time, and type) principles have not been discussed [14, 20]. It is essential for therapists to describe aerobic exercise in terms of FITT principles in order to titrate the appropriate dosage [29].

The primary aim of this review was to systematically evaluate the clinical (human) studies examining the effects of aerobic exercise on walking ability in MS. The second aim was to determine the aerobic exercise training parameters (FITT) that enhance both walking ability and proneuroplastic biomarkers (neurotrophins) in PwMS. The third aim was to analyze the extent to which aerobic exercise protocols evaluated in animal research can be translated into clinical practice.

## 2. Methods

### 2.1. Eligibility Criteria

Randomized clinical studies that evaluated the effects of aerobic/endurance-type exercise programs (swimming, walking, jogging, bicycling, treadmill, etc.) among PwMS for a duration of at least 3 weeks were eligible for this review. Studies with outcomes on walking ability (primary study outcome) evaluating spatiotemporal parameters and/or endurance with or without serum levels of neurotrophins (BDNF, NGF, NT3, and NT4) were included.

We also included randomized controlled studies in animal models of MS (experimental autoimmune encephalomyelitis (EAE) or cuprizone). Animal studies in which aerobic-type exercise (voluntary/forced treadmill, wheel running, swimming, etc.) was evaluated for its effects on gait and neurotrophins in the blood/muscle/brain/spinal cord, performed both before and after disease induction, were included.

The studies that evaluated slow-paced exercise or combination training with low aerobic workload (yoga, tai chi, memory tasks, resistance training, etc.) were excluded. Only English language articles were included.

### 2.2. Search Strategy

**Figure 1:**
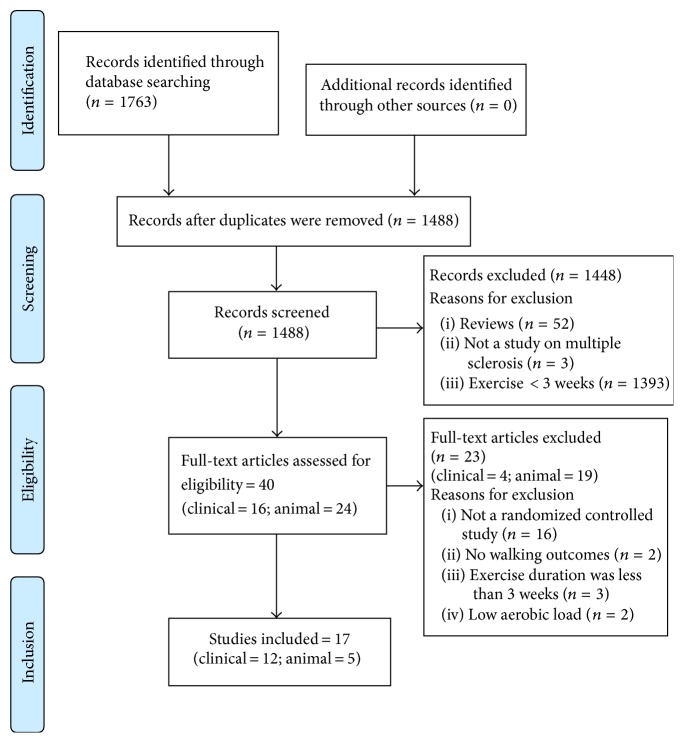
Flow chart - Systematic search strategy.

A systematic literature search was conducted in PubMed, EMBASE, Cochrane, Scopus, and Physiotherapy Evidence Database (PEDro), using a combination of keywords (multiple sclerosis, aerobic exercise, nerve growth factor, neurotrophic factor, and walking) and MESH/EMTREE terms in the respective databases (online supplement a, in Supplementary Material available online at https://doi.org/10.1155/2017/4815958). Two authors screened and assessed the eligibility of each article separately. Review articles and eligible articles were hand-searched for relevant references. The search strategy is presented in Figure 1 as per the adapted Preferred Reporting Items for Systematic Reviews and Meta-Analyses (PRISMA) guidelines from Cochrane review updates [30].

### 2.3. Methodological Quality Assessment

The clinical studies (*n* = 12) included in this systematic review were assessed for methodological quality using the Physiotherapy Evidence Database (PEDro) scale criteria [31, 32]. The quality of the clinical studies was classified as good for PEDro scores ≥ 6, fair for 4-5, and poor for ≤3 [31, 32]. These categories were selected based on previous research that conducted sensitivity analyses comparing results with cut-offs set at PEDro scores 4 to 6 [31, 32]. The animal studies (*n* = 5) were assessed for methodological quality using the SYstematic Review Centre for Laboratory animal Experimentation (SYRCLE) risk of bias tool, an adapted version of the Cochrane risk of bias tool developed for clinical studies [33].

### 2.4. Data Extraction and Analysis

Studies that compared the outcomes on walking ability (spatiotemporal parameters and/or endurance) between aerobic-type exercise and non-aerobic-type exercise or wait-list control were included for meta-analysis. The data, where available, from long walking tests that assessed endurance (2-minute and 6-minute walk tests) and short walking tests (10-meter walk test (mWT), functional ambulation profile (FAP) from GAITrite walkway) that assessed spatiotemporal parameters of walking were subjected to meta-analysis as previously performed by Miller et al. [34]. A strong association between 2-minute and 6-minute walk test results provided us with the justification to combine the data from these two long walking tests [35]. While both 10 mWT and FAP calculated by the GAITrite software are short walking tests measuring self-selected walking speed, the latter is a composite score integrating values of preferred walking speed and biomechanically related spatiotemporal walking parameters. This provided us with the rationale to combine the results from these two short walking tests. The data from studies reporting on energy cost (oxygen consumption in mL/kg/min) of walking were also included for analysis in a separate group.

The mean scores measured after the intervention period in experimental and control groups were used to calculate effect sizes (*d*). The sign of mean scores was reversed, where needed, to ensure all scores were aligned such that positive values on forest plot (right to the vertical line) favored improvements on walking ability due to aerobic-type interventions and the negative values on forest plot (left to the vertical line) favored wait-list control group or non-aerobic-type intervention. The standardized mean differences were calculated, as the outcomes pooled together in a group had different units of measure. The benchmark proposed by Cohen was used to describe small (*d* = 0.2), moderate (*d* = 0.5), and large (*d* = 0.8) effects of aerobic exercise on walking ability [36]. The chi-squared (*Q*^2^) value and *I*^2^ index were calculated to measure heterogeneity and inconsistency, respectively, among the studies included for meta-analysis.

## 3. Results

In total, 12 clinical studies and 5 animal studies were included in this review.

### 3.1. Methodological Quality Results

The methodological and reporting quality of the selected clinical studies is summarized in Table 1. Only 5 out of 12 clinical studies mentioned intention-to-treat analysis. None of the clinical studies reported blinding of subjects/therapists. The mean score of PEDro was 5.5 (SD: 0.9, range: 4–7) for 12 clinical studies. The quality of the clinical studies according to the total PEDro scores was good in 7 studies and fair in 5 studies. None of the clinical studies were of poor quality as per PEDro scores.

The methodological quality of animal studies included in this review is summarized in Table 2. None of the studies concealed the allocation of animals, randomly housed the animals, blinded the investigators and outcome assessors, or selected the animals randomly for outcome assessment (Table 2). The mean SYRCLE score was 4 (SD: 0.7, range: 3–5) for 5 animal studies. We note that it is still not standard practice to randomize treatment allocation or blind investigators and outcome assessors in animal research. We calculated SYRCLE score for each animal study to highlight methodological gaps and overall poor reporting quality. It is, however, not recommended to grade the quality of these studies (as good, fair, and poor) using summary scores for each study as this will require assigning “weights” to specific domains in the tool, which in turn will be difficult to justify [33].

### 3.2. Summary of Clinical Studies

We identified twelve clinical studies that evaluated the effects of aerobic training on walking outcomes (walking endurance and the spatiotemporal parameters of gait). Data on the FITT parameters and the outcomes on walking ability in the clinical studies are presented in Table 3. Five studies examined treadmill-training protocols [37, 42, 46–48]; three studies tested leg cycling protocols [40, 43, 44]; one study compared rowing and arm and leg cycling training [45]; two studies evaluated a combination of aerobic and strengthening exercises [39, 41]; and one study evaluated a calisthenics protocol [38].

**Figure 2:**
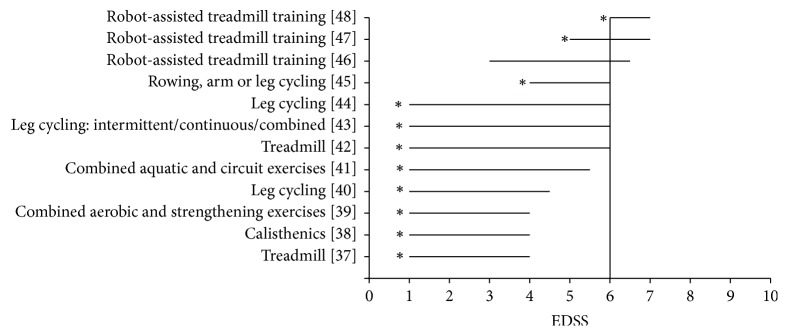
Aerobic interventions for varying disability levels. *x*-axis: the Expanded Disability Status Scale (EDSS) score ranges from no disability (0) to death (10). At 6.0, patients use walking aids. *y*-axis: the aerobic exercise interventions of experimental groups in the clinical studies included in this review. ^*∗*^Statistically significant improvements on walking performance.

Of these twelve studies, eleven reported significant improvements in walking ability (Figure 2). Among these eleven studies reporting recovery of walking, eight studies reported improvements on walking endurance (distance covered in a fixed time, time taken to cover a fixed distance—variables that represent a change on an individual's aerobic walking capacity) and eight studies reported improvements on spatiotemporal parameters of walking (biomechanical efficiency, namely, step length, stride length, cadence, single leg support time, and velocity) (Table 3). In total, we identified five types of aerobic interventions that improve walking ability: treadmill training, robot-assisted treadmill, cycling, calisthenics, and progressive repetitive endurance/strengthening activities (Figure 2). Only three studies investigated the effectiveness of an aerobic-type intervention on PwMS having severe difficulty walking (Figure 2) [46–48].

### 3.3. Effects of Aerobic Exercise Training on Walking Ability

Data from the studies that measured the effects of aerobic-type exercise on spatiotemporal walking parameters (10 mWT and FAP scores) showed a statistically significant improvement in walking ability (SMD = 0.83 [confidence interval (CI): 0.16, 1.50], *p* = 0.01, *I*^2^ = 28%) favoring aerobic exercise. Pooling together two studies that measured the effects of aerobic-type exercise on walking endurance (2-minute and 6-minute walk test scores) showed a trend favoring aerobic exercise (SMD = 0.59 [CI: −0.14, 1.32], *p* = 0.11, *I*^2^ = 0%). The outcomes on energy cost of walking also showed a trend favoring aerobic exercise (SMD = 0.65 [CI: −0.03, 1.32], *p* = 0.06, *I*^2^ = 0%). The participants in the studies included for meta-analysis [37, 42, 44] had mild to moderate walking impairments (EDSS: 1 to 6). Overall, there is a large effect of aerobic-type exercise on improving walking ability (spatiotemporal parameters) in people having mild–moderate walking impairments. Please refer to online supplement b for a forest plot on walking outcomes from the clinical studies included for meta-analysis. All other outcomes on walking and neurotrophins in both clinical and animal studies were not included for meta-analysis due to the lack of comparison with a control group intervention having lower exercise workload or varied responsiveness of the outcome measures with similar constructs [56].

### 3.4. Retention of Gains after the End of Aerobic Intervention

**Figure 3:**
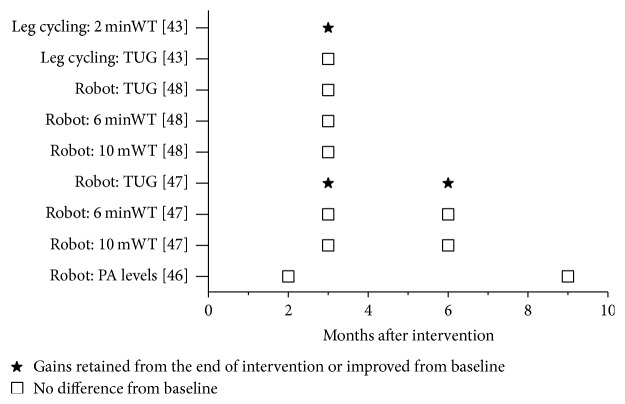
Summary of follow-up assessment findings after the end of aerobic exercise interventions. *X*-axis: time of follow-up assessments (in months). *Y*-axis: walking ability outcomes in the studies that had follow-up assessments. minWT: minute walk test; mWT: meter walk test: TUG: timed up and go test (in secs); Robot: robot-assisted treadmill training; PA: physical activity.

In total, only four of the twelve studies evaluated the retention of training effects after the conclusion of aerobic intervention (Figure 3) [43, 46–48]. Among these, two studies found no difference in walking ability from baseline [46, 48] and two studies reported mixed results [43, 47] (Figure 3). In those with mixed findings, a study on leg cycling reported gains retained from the end of intervention on the 2-minute walk test but reported detraining on timed up and go (TUG) results during their follow-up assessment [43], and a study on robot-assisted treadmill training reported improved TUG results but no difference from baseline on 6-minute and 10-meter walk tests [47] (Figure 3).

### 3.5. Exercise Methods That Improve Walking Ability

Our results indicate that most aerobic inventions that utilize the reciprocal motion of walking (task-specific training; [37, 42, 46–48]) as well as those that do not [38–41, 43–45] improve walking ability. Two studies that investigated treadmill (gait-specific) training reported improvements in both walking domains (endurance and spatiotemporal parameters) [37, 42] (Table 3). Studies on robot-assisted treadmill training (*n* = 3) reported varied results, with one study having no improvements in both walking domains compared to over-ground walking training [46] and the other two studies reporting improvements on TUG and 6-minute walking endurance, respectively, compared to conventional walking therapy (Table 3) [47, 48].

There were conflicting findings in the studies that provided aerobic exercise without gait training such as leg/arm cycling, calisthenics, and combined endurance and resistance training. One study that evaluated leg cycling reported improvements in 6-minute walking endurance but not in the cost of walking (mL O_2_/kg/m) [44]. Another study that evaluated three different cycling protocols reported improvement in TUG after the first 6 weeks of intervention but showed reversal of training effects during the 3-month follow-up assessment [43], and lastly, a study on leg cycling reported improvements in figure-of-8 walking but not in 3-meter walking coordination [40] (Table 3).

**Figure 4:**
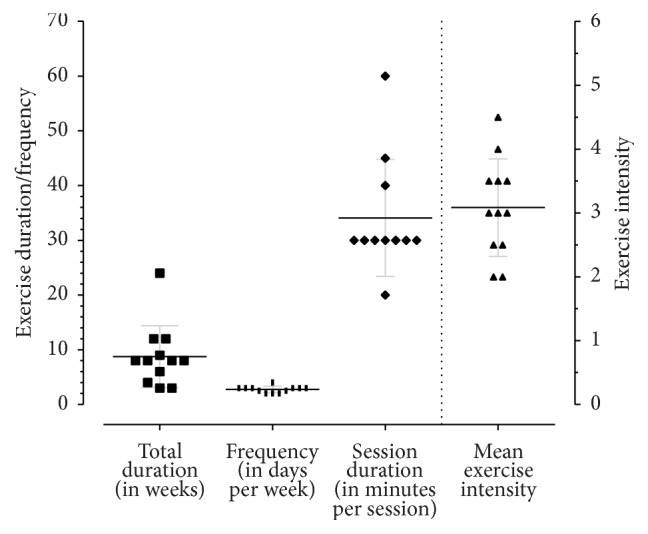
Summary of exercise parameters. *X*-axis: total duration of exercise program (in weeks), frequency of exercise sessions (number of days per week), duration of exercise sessions (in minutes per session), and intensity of exercise sessions in each study included in this review. Left *Y*-axis: exercise duration and frequency. Right *Y*-axis: exercise intensity (1: very light, 2: light, 3: moderate, 4: hard, 5: very hard, and 6: maximum) (adapted from ACSM's guidelines for exercise testing and prescription, 9th edition, 2013) [55]. The measures of dispersion (mean and standard deviations) of exercise parameters are indicated by the horizontal lines transecting the data points.

We summarized the findings in Table 3 to identify the parameters of exercise that improve walking endurance and spatiotemporal parameters separately.  Figure 4 presents the duration, frequency, and intensity of aerobic-type exercise programs (experimental group) evaluated in the studies included in this review. The exercise parameters that improved walking ability were as follows:

  Frequency: three times per week for at least 6–8 weeks
  Intensity: 40–75% age predicted maximum heart rate or 30–60% work rate for those with low to moderate levels of disability (EDSS < 6); maximum walking speed tolerated for people with higher levels of disability (EDSS ≥ 6)
  Time: at least 30 minutes per session
  Type: aerobic-type training on a treadmill (EDSS < 6)/leg cycling (EDSS ≤ 6)/game based or combined aerobic and strengthening exercise (EDSS < 6)/calisthenics (EDSS < 4.5)/robot-assisted treadmill (EDSS 5–7)

### 3.6. Exercise Methods That Improve Both Walking Ability and Neuroplastic Outcomes

We identified eleven clinical studies that reported significant improvements in walking outcomes, out of which two measured both walking and serum levels of neurotrophins [40, 45].

In the study by Schulz et al. [40], aerobic-type leg cycling for 8 weeks (30 min/session, twice a week, at 75% max. watts intensity) improved walking ability as measured using a figure-of-8 walking test. A significant decrease in lactate levels (before: 2.5 ± 1.8; after: 2.1 ± 2.3 mmol/l) was noted after a 30-minute endurance test after the intervention; however, there were no statistically significant pre-to-post changes in resting BDNF, NGF, IL-6, sIL-6R, ACTH, cortisol, epinephrine, or norepinephrine levels in the blood. This suggests that increased aerobic fitness (improved lactate response) achieved through leg cycling did not influence resting levels of neurotrophins among PwMS. However, there was an increase of BDNF in the training group (descriptively) while levels in the control group decreased. This was noted on both resting levels and acute response to 30-minute endurance test.

In the study by Briken et al. [45], walking endurance was assessed before and after 22 sessions of interval-type aerobic rowing/arm/leg cycling (2-3 sessions/week, for 9 weeks; stepwise progression of intensity). No association between the change scores of 6-minute walk distance and BDNF was found considering all 3 intervention groups together [45]. However, they found an increase in the 6-minute walk distance after intervention (arm/leg cycling groups) in their pilot work [54]. The authors noted a nonsignificant increase in the resting BDNF levels after 22 training sessions and attributed the reason for nonsignificance to small sample size [45].

**Figure 5:**
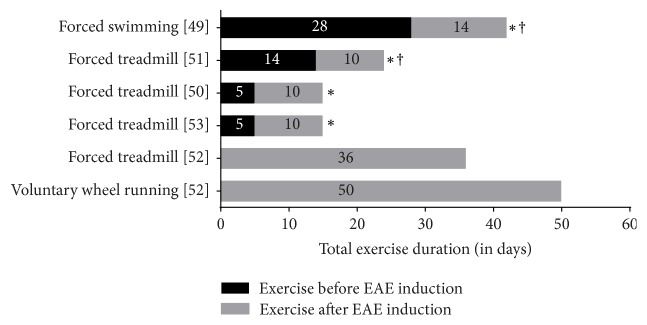
Summary of the results of aerobic exercise interventions in animal models of MS. *X*-axis: total number of days exercised by the animals in the experimental group in the animal studies included in this review. *Y*-axis: aerobic exercise interventions in the experimental groups. ^*∗*^Improvements in neurotrophic markers. ^†^Improvements in disease status or gait outcomes.

There is not enough data to extrapolate our findings and suggest optimal exercise parameters that could improve walking and upregulate neurotrophins. However, based on two clinical studies [40, 45, 54], the FITT parameters that improved walking ability with a trend towards an increase in neurotrophins were as follows:

  Frequency: 2 to 3 times per week for at least 8 to 9 weeks
  Intensity: light to hard (Figure 5), interval-type training and stepwise progression of intensity with similar total workload
  Time: at least 30 minutes per session
  Type: aerobic-type leg cycling (EDSS < 6)

### 3.7. Summary of Animal Studies with Outcomes on Gait and Neurotrophins

We identified 5 studies that investigated the effects of aerobic exercise on neurological status and neurotrophins in animal models of MS (online supplement c). Only two studies showed significant improvements in neurological status and both instituted exercise for 2 weeks or more* before* EAE induction [49, 51] (Figure 5). Four out of five studies reported a significant change with exercise on the levels of neurotrophins (BDNF or NGF) in the brain (*n* = 2), spinal cord (*n* = 1), serum (*n* = 1), and muscle (*n* = 1) (online supplement c). All of these studies also initiated exercise* before* induction of EAE. In one study [41], although there was no difference in hippocampal BDNF between sedentary and exercising (forced treadmill, voluntary wheel running) mice, higher amounts of exercise were positively correlated with a higher concentration of hippocampal BDNF [52] (Figure 5).

In animal models of MS, FITT parameters that most consistently improved both neurotrophins and neurological outcomes were as follows:

  Frequency: daily exercise for at least 14 days before induction of EAE
  Intensity: at least 60% maximum workload or 55% maximal oxygen consumption
  Time: at least 30 to 60 minutes per session/day
  Type: forced aerobic-type treadmill running or swimming

## 4. Discussion

The American College of Sports Medicine (ACSM) [55] recommends 10–60 minutes of progressive aerobic exercise at an intensity of about 40%–70% oxygen consumption reserve or heart rate reserve ranging between 11 and 14 levels on a rate of perceived exertion (RPE) score for 3–5 days per week, in order to maximize health and fitness benefits for PwMS. However, these exercise recommendations are designed to address cardiorespiratory fitness and not walking impairments and neuroplasticity.

In this review, we sought to identify the optimal type of aerobic exercise and training parameters that could lead to improvements in walking ability in PwMS and promote brain repair through the upregulation of neurotrophic factors. We report five key findings: (1) the clinical studies were of fair to good quality and consistently showed that aerobic interventions (ranging from mild to vigorous intensities) improved walking endurance and spatiotemporal parameters of gait in people with EDSS scores less than 6 (able to walk independently); interventions that did not employ the reciprocal motions of walking (i.e., which were not task-specific) improved walking endurance more consistently than they did for the spatiotemporal parameters; (2) very few studies examined whether effects were sustained after cessation of the intervention, and those that did showed that most outcomes return to baseline within a few months; (3) people with severe MS-related walking impairments (EDSS 6 and above) were relatively underrepresented in the studies; (4) in clinical studies, neurotrophins were not reliably changed with aerobic exercise; (5) in animal studies, both neurotrophins and neurological status were improved when aerobic exercise began more than 2 weeks* before* the induction of EAE in the animal.

### 4.1. Aerobic Exercise with or without Gait-Specific Training

Our findings from 12 clinical studies suggest that aerobic exercise targeting the reciprocal movements of gait per se is not required in order to improve walking in MS. Participants also had improved walking endurance and walking quality with nongait activities such as leg/arm cycling, swimming, and calisthenics. Physical therapists, therefore, can use multiple aerobic exercise modalities to affect gait. This is particularly important for home-based and community-based exercise which may make use of arm cycling or swimming. Our findings are similar to those in chronic neurological conditions like stroke, cerebral palsy, and Parkinson's disease [57–60] which showed that multiple methods can be employed with similar benefits in walking. For example, Nadeau et al. [57, 58] reported from their LEAPS trial that both task-specific locomotor training and impairment-based home exercises were equally effective in improving comfortable/fast walking speed as well as 6-minute walking distance in stroke. Kumar and Ostwal [59] compared the effects of task-oriented training and proprioceptive neuromuscular facilitation exercises in children with cerebral palsy having difficulty walking and reported improved gait velocity with no difference between the two groups. Similarly, Shulman et al. [60] found that treadmill and resistance training did not differ in improving gait among people with Parkinson's disease.

### 4.2. Sustainability of the Benefits of Aerobic Training

Only four of the 12 studies examined whether improvements were sustained after cessation of the training program and most showed that outcomes return to baseline within a few months (Figure 3). It is not clear whether participants stopped exercising after cessation of the study or whether there was deterioration in the disease during the follow-up period. Our exercise recommendations may not result in neuroplasticity of walking as we did not observe long-term restoration of function in the clinical studies included in this review. In some cases, especially in more progressive diseases, maintaining the baseline is considered a positive outcome. For example, among people with chronic incomplete spinal cord injury (a more stable neurological condition), thrice weekly body weight supported treadmill training for one year resulted in retention of gains up to 8 months after the end of the intervention [61]. Future studies, in addition to measuring outcomes at follow-up, should also record physical activity levels (accelerometry) to determine whether newly gained skills are being incorporated into everyday activities. Interventions should also be designed such that they could be continued at home or in the community and the benefits are sustained [62].

### 4.3. Underrepresentation of People Having Gait Impairments

It is important that research undertaken to improve gait include people with MS who have problems with walking. Eight of the 12 studies included participants who had EDSS scores less than 3 and even EDSS 1, suggesting very minimal impairment levels (Figure 2). Clearly, more research is required to determine whether walking outcomes can be changed in PwMS who have already acquired walking difficulties. The results of interventions using robot-assisted treadmill were promising [47, 48]. Although Vaney et al. [46] noted clinical benefits to practice walking over ground compared to robot-assisted treadmill, a high volume of training and high walking impairment (slow walking speed) could be the determining factors for success using robot-guided treadmills. In order to tailor aerobic interventions for those with higher degrees of walking impairment, it would be prudent to involve patients as partners and consultants in the research process in order to meet their needs [63].

### 4.4. Need for Novel Exercise Strategies

People having an MS-related disability often report higher rates of exercise-induced fatigue [64]. Future research should focus on investigating strategies to increase the tolerance to vigorous intensity aerobic training load without increasing the training side effects such as fatigue. An example of such a strategy will be to conduct high-intensity interval training using basic functional tasks (getting up from bed, sit to stand, and walking) for those with high MS-related disability as it may be more effective in optimizing recovery than performing continuous training at similar total workload.

### 4.5. Translating Research from Animal Models to the Clinical Condition

We aimed to examine the findings in animal studies to determine their applicability to MS clinical research. Of the five studies examining aerobic exercise in an animal model of MS (EAE), exercise benefited walking and increased neurotrophins only when instituted two weeks or more before EAE induction. This suggests that aerobic exercise is likely neuroprotective but provides little benefit when employed after MS is induced in the animal. The neuroprotective effects of exercise have been reported in animal models of ischemic stroke [65] in which exercise enhanced neurogenesis, angiogenesis, and synaptogenesis [66] possibly providing redundancy and tolerance to subsequent injury. The findings reported in this review may support the notion that exercise may be able to reduce the impact of MS relapse rather than altering the outcome after relapse. A major caveat to translating findings is that the animal studies report neuroprotective effects of aerobic exercise, whereas clinical studies have found positive benefits of aerobic exercise following MS. Clearly, more research is required to disentangle the timing, duration, and intensity of exercise before and after MS relapse.

### 4.6. BDNF Upregulation and Neuroplasticity of Walking

We also showed that, with only two clinical studies and four animal studies examining BDNF as a potential biomarker of plasticity, the results are inconclusive on whether serum levels of BDNF indicated exercise-related repair of the CNS. However, a recent meta-analysis of 13 studies on a mixed population (80 MS patients out of a total of 703 patients) showed increased magnitude of BDNF responsivity and higher resting levels of BDNF after exercise training [67]. Further research examining both resting and exercise-induced levels of BDNF is needed to elucidate the relationship between plasticity and neurotrophins in MS. Additionally, it is important to consider the influence of factors such as sex, genetics, nutrition, smoking, and other confounders while examining the impact of exercise on BDNF [68].

## 5. Conclusion

Consolidated evidence suggests that aerobic exercise training can improve walking ability (spatiotemporal walking parameters) in people having MS without severe walking impairments. In this review, we have outlined the optimal aerobic training parameters (30 min 3x week for 6–8 weeks at mild to vigorous intensity) that improved walking in people with EDSS scores less than 6.0 (able to walk independently). Although individual studies reported that gait-specific and non-gait-specific types of aerobic exercise improved both endurance and spatiotemporal parameters of walking, the effects of the aerobic exercise were not sustained more than six months after the end of intervention. There is a need to build exercise programs for people living with MS having higher disability, especially EDSS 6.0 or above, to restore their lost ability to walk.

In PwMS, the serum levels of neurotrophins measured at rest did not significantly change after completing a course of aerobic training. In contrast, the animal studies showed a significant change in both neurological recovery and neurotrophins in blood, muscle, and nervous tissue, especially when aerobic exercise begins 2 weeks before EAE induction.

## Supplementary Material

Search strategy and further details of included studies.

## Figures and Tables

**Table 1:** Methodological quality assessment of the clinical studies included in this review.

Articles included	PEDro scoring criteria											PEDro Score^*∗*^
	(1)	(2)	(3)	(4)	(5)	(6)	(7)	(8)	(9)	(10)	(11)	
Ahmadi et al. [37]	Y	Y	N	Y	N	N	N	N	N	Y	Y	4/10
Aydin et al. [38]	Y	Y	N	Y	N	N	N	Y	N	Y	Y	5/10
Dettmers et al. [39]	Y	Y	Y	Y	N	N	N	Y	N	Y	Y	6/10
Schulz et al. [40]	N	Y	N	Y	N	N	N	Y	N	Y	Y	5/10
Romberg et al. [41]	Y	Y	N	Y	N	N	N	Y	Y	Y	Y	6/10
Brændvik et al. [42]	Y	Y	N	Y	N	N	N	Y	Y	Y	Y	6/10
Collett et al. [43]	Y	Y	N	Y	N	N	Y	N	Y	Y	Y	6/10
Rampello et al. [44]	Y	Y	N	Y	N	N	Y	N	N	Y	Y	5/10
Briken et al. [45]	N	Y	N	Y	N	N	N	N	N	Y	Y	4/10
Vaney et al. [46]	Y	Y	Y	Y	N	N	N	N	Y	Y	Y	6/10
Schwartz et al. [47]	Y	Y	Y	Y	N	N	Y	Y	N	Y	Y	7/10
Straudi et al. [48]	N	Y	N	Y	N	N	Y	N	Y	Y	Y	6/10
Total score	9	12	3	12	0	0	4	6	5	12	12	

(1) Eligibility criteria; (2) random allocation; (3) concealed allocation; (4) baseline comparability; (5) blind subjects; (6) blind therapists; (7) blind assessors; (8) adequate follow-up; (9) intention-to-treat analysis; (10) between-group comparisons; (11) point estimates and variability. ^*∗*^The eligibility criteria item in the PEDro scale does not contribute to the PEDro score. Y (yes) = 1; N (no) = 0. PEDro: Physiotherapy Evidence Database; *n*: sum of scores; %: percentage.

**Table 2:** Methodological quality assessment of the animal studies included in this review.

Articles included	SYRCLE's risk of bias tool, scoring criteria										SYRCLE's score
	(1)	(2)	(3)	(4)	(5)	(6)	(7)	(8)	(9)	(10)	
Bernardes et al. [49]	N	Y	N	N	N	N	N	N	Y	Y	3/10
Patel and White [50]	Y	Y	N	N	N	N	N	N	Y	Y	4/10
Wens et al. [51]	Y	Y	N	N	N	N	N	Y	Y	Y	5/10
Klaren et al. [52]	Y	Y	N	N	N	N	N	N	Y	Y	4/10
Patel et al. [53]	Y	Y	N	N	N	N	N	N	Y	Y	4/10
Total score	4	5	0	0	0	0	0	0	5	5	

(1) Sequence generation; (2) baseline characteristics; (3) allocation concealment; (4) random housing; (5) blinding—investigators; (6) random outcome assessment; (7) blinding—outcome assessors; (8) incomplete outcome data addressed; (9) no selective outcome reporting; (10) no other sources of bias. Y (yes) = 1; N (no) = 0; U (unclear) = 0. SYRCLE: SYstematic Review Centre for Laboratory animal Experimentation; *n*: sum of scores; %: percentage.

**Table 3:** Outcomes on walking ability and neurotrophins from clinical studies.

Intervention	Pre-to-post changes in walking ability^‡^		Changes in walking ability during follow-up assessments^‡^	Pre-to-post changes in neurotrophins^‡^
	Walking endurance	Spatio-temporal parameters		
Treadmill^†^ versus yoga [37]	↑ 2 minWT (m)^*∗*^	↓ 10 mWT (m/s)^*∗*^	NT	NT

Calisthenics, hospital-based^†^ versus home-based [38]	NT	↓ 10 mWT (m/s)^*∗*^	NT	NT

Combined aerobic and strengthening exercises^†^ versus combined stretching, balance, and coordination exercises [39]	↑ Self-paced walking distance on treadmill^*∗*^↑ Walking duration on treadmill^*∗*^↑ Relative walking ability (time and distance)^*∗*^	NT	NT	NT

Leg cycling^†^ versus wait-list control [40]	NT	↑ Figure-of-8 left^*∗*^/right^*∗*^ walking coordination ^*⦰*^3 m walking coordination score	NT	^*⦰*^BDNF, ^*⦰*^NGF

Combined aquatic aerobic and circuit resistance exercises^†^ versus no intervention [41]	↓ 500 m walking time (min)^*∗*^	↓ 7.62 m (25 feet) walking time (secs)^*∗*^	NT	NT

Treadmill versus strength training [42]	↓ Oxygen uptake while walking: improved work economy^*∗*^	↑ Functional ambulation profile score^*∗*^↓ Root mean square of vertical acceleration^*∗*^	NT	NT

Leg cycling^†^: continuous versus combined versus intermittent [43]	↑ 2 min walk distance^*∗*^ (considering all participants together at 6 weeks during 12-week-long intervention) Post hoc analysis on 2 min walk distance revealed that the higher-intensity intermittent exercise group would have shown significantly greater improvements in walking mobility if the study had been powered with a sample size of 123	↓ TUG^*∗*^ (secs) from 0 to 6 weeks ^*⦰*^TUG (secs) from 6 to 12 weeks during 12-week-long intervention	^*⦰*^No changes in 2 min walk distance between post and 3-month follow-up ↑ TUG^*∗*^ (secs) between post and 3-month follow-up	NT

Leg cycling^†^ versus neurologic rehabilitation [44]	↑ 6 minWT distance^*∗*^^*⦰*^Cost of walking (mL O_2_/kg/m)	↑ Walking speed (m/min)^*∗*^	NT	NT

Rowing, arm or leg cycling^†^ versus wait-list group [45]	^*⦰*^Considering all intervention groups together, there is no association between 6 min walk test and BDNF change scores [45] ↑ 6 min WT (arm/leg cycling)^*∗*^ reported in the pilot randomized trial [54]	NT	NT	^*⦰*^No association between the change scores of BDNF and 6 min walk test [45] ^*⦰*^No change in resting serum BDNF levels after 22 training sessions [45]

Robot-assisted treadmill training^†^ versus over-ground walking [46]	^*⦰*^3 minWT (m/s)	^*⦰*^10 mWT (m/s)	^*⦰*^No change between baseline and post, 2nd-, and 9th-month follow-up on movement counts and mins of physical activity over 3 METs on an accelerometer	NT

Robot-assisted treadmill training^†^ versus conventional walking treatment [47]	^*⦰*^6 minWT distance	^*⦰*^10 mWT (m/s) ↓ TUG (secs)^*∗*^	Change between baseline and 3rd- and 6th-month follow-up in TUG (secs)^*∗*^^*⦰*^No change from baseline on 6 minWT and 10 mWT	NT

Robot-assisted treadmill training^†^ versus conventional walking therapy [48]	↑ 6 minWT distance^*∗*^	^*⦰*^10 mWT (m/s) ^*⦰*^TUG (secs)	^*⦰*^No change between baseline and 3-month follow-up in 6 minWT, 10 mWT, and TUG scores	NT

^†^Aerobic-type intervention in the experimental group; ^‡^results from the experimental group; ^*∗*^significance at *p* < 0.05; ^*⦰*^changes not significant; NT: not tested; m: meter; min: minute; secs: seconds; m/s: meters per second; ft: feet; BWS: body weight support; WT: walk test; TUG: timed up and go; MFU: month follow-up; RAGT: robot-assisted gait training; BDNF: brain derived neurotrophic factor; NGF: nerve growth factor; METs: metabolic equivalents.

## Additional Points

*Limitations*. There are some limitations in this review. First, despite a carefully conducted search strategy, we cannot be sure that all studies were identified. Second, we did not include articles published in languages other than English. Third, because of the diversity of interventions and outcomes, we were unable to include data from all selected studies in our meta-analysis. As more research emerges, calculation of effect sizes using actual mean differences would be clinically useful. Fourth, both clinical and animal studies included in this review had methodological gaps (Tables 1 and 2). For example, none of the clinical studies blinded therapists and none of the animal studies ensured allocation concealment or random housing of animals. Clearly, there is a need for high-quality research in the field of aerobic exercise interventions to improve walking in MS.
