# Supplementary material for: The Effects of Aerobic Exercise on the Recovery of Walking Ability and Neuroplasticity in People with Multiple Sclerosis: A Systematic Review of Animal and Clinical Studies
Source: Mult Scler Int. 2017 Oct 17;2017:4815958. doi: 10.1155/2017/4815958 (PMC5664281; doi:10.1155/2017/4815958)
Supplement: Supplementary file 1 — Search strategy and further details of included studies. [file 4815958.f1.pdf]

ONLINE SUPPLEMENTS

a. Search terms used in the systematic search strategy:

The following search terms were used for PubMed and adapted for Embase, Cochrane, Scopus and PEDro to find clinical studies:

#1 (("Multiple Sclerosis"[Mesh]) OR ("multiple sclerosis"[tiab]))

#2 (("Exercise"[Mesh]) OR (aerobic OR exercise OR swim OR swimming OR walk OR walking OR jog OR jogging OR run OR running OR bicycle OR bicycling OR dance OR dancing))

#3 #1 AND #2

#4 ((“Nerve Growth Factors”[MeSH Terms]) OR (Brain Derived Neurotrophic Factor OR Nerve Growth Factor))

#5 #3 AND #4

The following search terms were used for PubMed and adapted for Embase, and Scopus to find animal studies:

#1 (Experimental Autoimmune Encephalomyelitis[MeSH Terms])

#2 (“Exercise”[MeSH Terms])

#3 #1 AND #2

b. Forest plot of comparison for walking ability outcomes between aerobic exercise and wait-list control or non-aerobic exercise from clinical studies:

	Experimental			Control				Std. Mean Difference
Study or Subgroup	Mean	SD	Total	Mean	SD	Total	Weight	IV, Random, 95% CI
**1.1.1 Spatio-temporal Walking Parameters**								
Aerobic Cycling vs Neuro Rehab [Rampello 2007]	55	18	6	51	18	5	24.9%	0.20 [-0.99, 1.39]
Treadmill vs Strengthening [Braendvik 2015]	94.2	3.7	11	90.3	6.2	15	43.5%	0.71 [-0.09, 1.52]
Treadmill vs Wait-list Control [Ahmadi 2013]	-7.07	1.03	10	-9.47	1.92	10	31.6%	1.49 [0.47, 2.51]
**Subtotal (95% CI)**			**27**			**30**	**100.0%**	**0.83 [0.16, 1.50]**
Heterogeneity: Tau² = 0.10; Chi² = 2.77, df = 2 (P = 0.25); I² = 28%								
Test for overall effect: Z = 2.44 (P = 0.01)								
**1.1.2 Walking Endurance**								
Aerobic Cycling vs Neuro Rehab [Rampello 2007]	332	108	6	308	110	5	37.5%	0.20 [-0.99, 1.39]
Treadmill vs Wait-list Control [Ahmadi 2013]	139.9	20.78	10	119.05	27.12	10	62.5%	0.83 [-0.10, 1.75]
**Subtotal (95% CI)**			**16**			**15**	**100.0%**	**0.59 [-0.14, 1.32]**
Heterogeneity: Tau² = 0.00; Chi² = 0.66, df = 1 (P = 0.42); I² = 0%								
Test for overall effect: Z = 1.59 (P = 0.11)								
**1.1.3 Energy Cost of Walking**								
Aerobic Cycling vs Neuro Rehab [Rampello 2007]	-0.2	0.07	6	-0.22	0.09	5	31.9%	0.23 [-0.96, 1.42]
Treadmill vs Strengthening [Braendvik 2015]	-11.38	1.33	11	-12.66	1.57	15	68.1%	0.84 [0.02, 1.66]
**Subtotal (95% CI)**			**17**			**20**	**100.0%**	**0.65 [-0.03, 1.32]**
Heterogeneity: Tau² = 0.00; Chi² = 0.69, df = 1 (P = 0.41); I² = 0%								
Test for overall effect: Z = 1.88 (P = 0.06)								

Test for subgroup differences: Chi² = 0.26, df = 2 (P = 0.88), I² = 0%

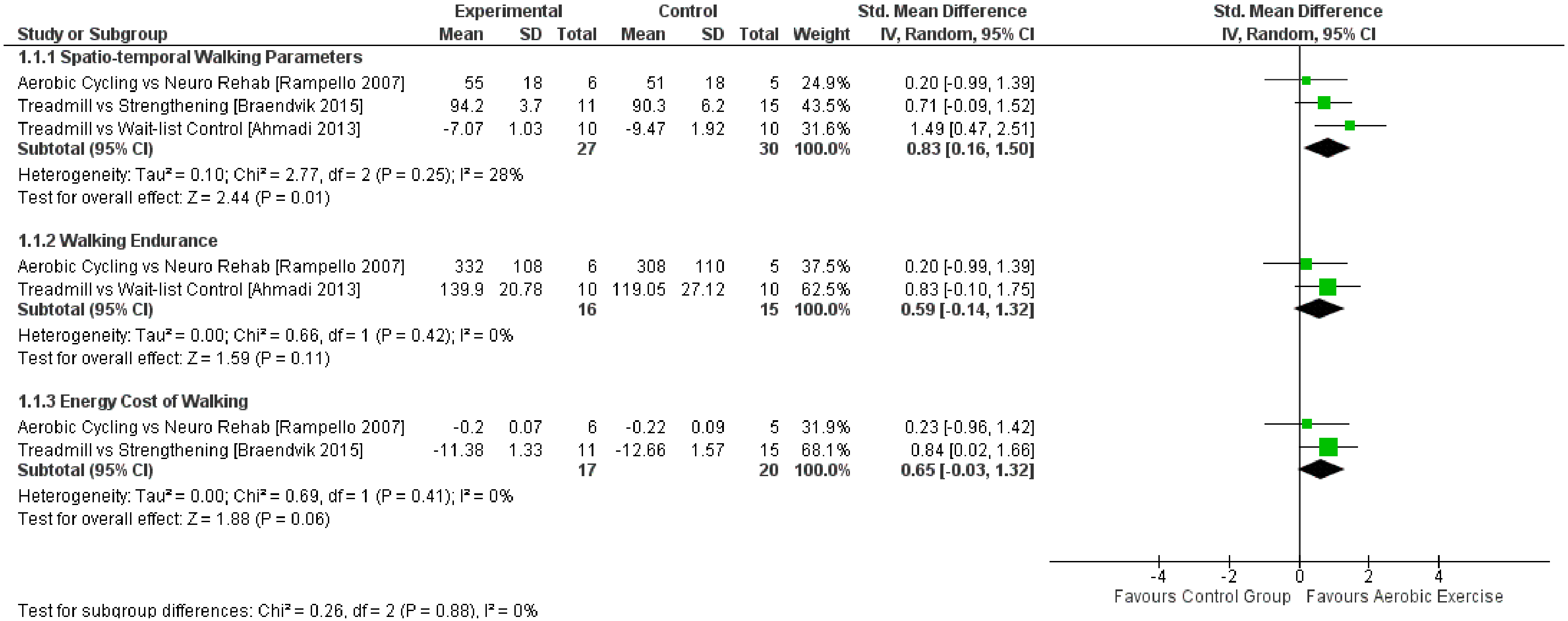

Figure a: Forest plot of comparison for walking ability (spatio-temporal parameters, endurance and energy cost of walking); Risk of Bias categories – A: random sequence generation (selection bias), B: Allocation concealment (selection bias), C: Blinding of participants and personnel (performance bias), D: Blinding of outcome assessment (detection bias), E: Incomplete outcome data (attrition bias), F: Selective reporting (reporting bias), G: Other bias; CI: confidence interval; df: degree of freedom; %: percentage; P = p value.

c. Table on summary of animal model studies included in this review:

Selected Trials	Aerobic Intervention†	Frequency/Intensity/Time	Neuroplastic blood/tissue markers	Disease status/ Gait outcomes
Bernardes et al (49)	Forced swimming	Progressive adaptation in swimming pool: days 1 to 4; followed by progressive load test on day 5; followed by training with intensity set at 60% maximum weight obtained in load test;<br>Before EAE induction: Swimming for 30 min/day, 5 days/week, for 4 weeks; After EAE induction: 10 days' post-induction, 30 min/day	Brain and spinal cord BDNF levels (in pg/mL) in both brain and spinal cord homogenates*	In exercising EAE mice: hind limb paralysis improved*; decreased weight loss*; delayed development of EAE signs*; demyelination in brain and spinal cord*
Patel et al (50)	Forced treadmill running	Habituation: 5 days, daily treadmill run progressing from 10 to 50 min at 55% maximal oxygen consumption at 0 grade;<br>Training: Rodents ran 60 mins on days 1-2 and 90 mins on days 3 -10 with an increasing intensity starting at 15 m/min for 30 mins then increased to 30 m/min for the remaining time	Whole brain concentrations of BDNF (in pg/g), NGF (in pg/g) in exercising EAE rodents*	No significant difference in clinical disability scores
Wens et al (51)	Treadmill running	Habituation: progressive increase in running duration and intensity over 2-week period using short electric shocks, until a running duration of 1 hour and a running speed of 18m/min (25° inclination) was reached; followed by EAE & treadmill running daily for 1 hr/day for 10 consecutive days	Serum BDNF (in pg/mL)*	Delayed peak disease occurrence in exercising mice*; No difference in peak disease severity between exercising and sedentary mice; The hindquarter paralysis score (1-5 scale) tended to improve (p=0.07) over time in exercising mice with no

				difference in the degree of recovery on the last day of experiment
Klaren et al (52)	voluntary wheel running, forced treadmill exercise, sedentary	Voluntary wheel running (housed in cages with running wheel – 50 days), or forced treadmill exercise (subjected to 5days/week of running on a motorized treadmill (DC5; Jog-aDog, Ottawa Lake, MI) at a 5% grade, 14 m/min, for 30 min for 36 days)	No significant effects of exercise delivered during remission after the initial disease onset levels of hippocampal BDNF (in pg/mg)	No change in clinical disability scores
Patel et al (53)	Forced treadmill running	As described by Patel et al (50)	BDNF and NGF concentrations in soleus (in pg/mL)*	No difference in onset of clinical disability, disability ↑ in exercising EAE mice*

[†]aerobic intervention in the experimental group; *significant results; &: and; CB1: cannabinoid receptor type 1; EAE: experimental auto-immune encephalomyelitis; BDNF: brain-derived neurotrophic factor; cFOS: a 380 amino acid protein; NR1: subunit of functional NMDA glutamate receptor; CD3+: a type I transmembrane protein found on T cells; Iba1: ionized calcium-binding adapter molecule 1; TNF: tumor necrosis factor; m: meter; min: minute; ET: endurance training; ST: strength training; pg: picogram; mL: milli-litre; mg: milli-gram
